# Interaction between electrical storm and left ventricular ejection fraction as predictors of mortality in patients with implantable cardioverter defibrillator: A Chinese cohort study

**DOI:** 10.3389/fcvm.2022.937655

**Published:** 2022-07-29

**Authors:** Zhengqin Zhai, Shuang Zhao, Xiaoyao Li, Keping Chen, Wei Xu, Wei Hua, Yangang Su, Min Tang, Zeyi Li, Shu Zhang

**Affiliations:** ^1^China-Japan Friendship Hospital, Beijing, China; ^2^Fuwai Hospital, Chinese Academy of Medical Sciences and Peking Union Medical College, Beijing, China; ^3^Nanjing Drum Tower Hospital, Nanjing, China; ^4^Zhongshan Hospital, Fudan University, Shanghai, China

**Keywords:** electrical storm, implantable cardioverter defibrillator, left ventricular ejection fraction, mortality, predictors

## Abstract

**Aims:**

To determine the interaction of electrical storm (ES) and impaired left ventircular ejection fraction (LVEF) on the mortality risk of patients with implantable cardioverter defibrillator (ICD).

**Methods and results:**

A total of 554 Chinese ICD recipients from 2010 to 2014 were retrospectively included and the mean follow-up was 58 months. The proportions of dilated cardiomyopathy and the hypertrophic cardiomyopathy were 26.0% (144/554) and 5.6% (31/554), respectively. There were 8 cases with long QT syndrome, 6 with arrhythmogenic right ventricular cardiomyopathy and 2 with Brugada syndrome. Patients with prior MI accounted for 15.5% (86/554) and pre-implantation syncope accounted for 23.3% (129/554). A total of 199 (35.9%) patients had primary prevention indications for ICD therapy. Both ES and impaired LVEF (<40%) were independent predictors for all-cause mortality [hazard ratio (HR) 2.40, 95% CI 1.57–3.68, *P* < 0.001; HR 1.94, 95% CI 1.30–2.90, *P* = 0.001, respectively] and cardiovascular mortality (HR 4.63, 95% CI 2.68–7.98, *P* < 0.001; HR 2.56, 95% CI 1.47–4.44, *p* = 0.001, respectively). Compared with patients with preserved LVEF (≥40%) and without ES, patients with impaired LVEF and ES had highest all-cause and cardiovascular mortality risks (HR 4.17, 95% CI 2.16–8.06, *P* < 0.001; HR 11.91, 95% CI 5.55–25.56, *P* < 0.001, respectively). In patients with impaired LVEF, ES increased the all-cause and cardiovascular mortality risks (HR 1.84, 95% CI 1.00–3.37, *P* = 0.034; HR 4.86, 95% CI 2.39–9.86, *P* < 0.001, respectively). In patients with ES, the deleterious effects of impaired LVEF seemed confined to cardiovascular mortality (HR 2.54, 95% CI 1.25–5.14, *p* = 0.038), and the HR for all-cause mortality was not significant statistically (HR 1.14, 95% CI 0.54–2.38, *P* = 0.735).

**Conclusion:**

Both ES and impaired LVEF are independent predictors of mortality risk in this Chinese cohort of ICD recipients. The interaction of ES and impaired LVEF in patients significantly amplifies the deleterious effects of each other as distinct disease.

## Introduction

Electrical storm (ES) is a strong predictor of mortality in patients with implantable cardioverter defibrillator (ICD). A meta-analysis of 5,912 patients from 13 clinical studies demonstrated that ES increased the risk of death significantly (risk ratio 3.15) (1). Impaired left ventricular ejection fraction (LVEF) is known to act as independent predictor of mortality in these patients as well (2). However, it should be noted that some studies described low LVEF as being associated with ES and could be used to define specific populations with higher risk to develop ES (3–6). Is has also been described that reduced LVEF and ES are both independent predictors for mortality in patients with ICD at the same time (7). However, whether ES is an independent causal factor or just an epiphenomenon of impaired LVEF in uncertain, and none of the studies were designed to correlate the effect of ES on mortality in patients with different LVEF, especially in patients with preserved LVEF (≥40%), and whether impaired LVEF (<40%) could increase the risk of mortality further in ICD recipients with ES.

The aims of our studies were as follows. First, we wanted to ascertain whether ES is associated with higher mortality in patients with ICD, and if so, whether the association between ES and mortality is modified by impaired LVEF. Secondly, we wanted to investigate the prognostic impact of impaired LVEF on mortality and whether its effect is modified by the presence of ES.

## Methods

### Patient population

We retrospectively and consecutively included a total of 554 patients who underwent ICD implantation between 1 May 2010 and 30 April 2014 from the Study of Home Monitoring System Safety and Efficacy in Cardiac Implantable Electronic Device-implanted patients (SUMMIT) registry in China. The protocols were approved by the hospital ethics committees, and all patients gave their informed consent at the time of enrollment. Patient baseline characteristics, including age, sex, comorbidities and medications, were collected at the time of ICD implantation. The inclusion criteria were (1) patients with an ICD (Biotronik, Berlin, Germany) equipped with home monitoring system that could process daily transmissions, and (2) patients with echocardiographic evaluations prior to implantation procedure.

### ICD settings and ES definition

The programming settings were as follows: the basic pacing rate was 40–60 bpm. The target VT monitor zone was 140–170 bpm, the VT therapy zone was over 170–210 bpm, and VF zone was over 210 bpm. In VT therapy zone, 2–3 bursts of anti-tachycardia pacing (ATP) were delivered and shock followed if ATP failed and episodes persisted. In VF zone, shock was used. The detection interval was 26 beats in VT zone with a 20-beat re-detection and the detection interval was 12 out of 16 beats in VF zone. The discrimination algorithm was Biotronik SMART^®^ algorithm. Other programmable parameters were at the discretion of individual physicians depending on the patients' condition.

### Groups of patients

Based on the presence or absence of ES, patients were divided into ES group and No-ES groups. Using the most recent LVEF as measured prior to the ICD implant, patients were divided into preserved LVEF group (≥40%) and impaired LVEF group (<40%). To study the interaction between ES and LVEF, there were further divided as follows (1) Group I: LVEF < 40% with ES; (2) group II: LVEF < 40% without ES; (3) group III: LVEF ≥ 40% with ES; (4) group IV: LVEF ≥ 40% without ES.

### Outcome measures

The outcomes measures included the incidences of all-cause and cardiovascular mortality. Cardiovascular death was defined as death due to heart failure, cardiogenic shock, sudden cardiac arrest, hemodynamically unstable arrhythmias and other cardiac reasons as diagnosed by the local hospital. In case of patient death, the data and cause of death was confirmed with their families based on the death certificate.

### Statistical analysis

Categorical variables are presented as numbers and percentages and were compared using the chi-square test or the Fisher exact test. Quantitative variables were checked for normality using the Kolmogorov-Smirnov test. Normally distributed variables are reported as mean ± SD and non-normally distributed variables are presented as median (interquartile range). Kaplan-Meier curves were plotted to evaluate the association between groups regarding clinical outcomes and differences were assessed using the log-rank test. Cox proportional hazards regression was used for univariable and multivariable survival analyses. Hazard ratio (HR) and 95% confidence interval (CI) were calculated for each variable for the endpoints. All variables that had statistically significant effect were introduced into a multivariable Cox proportional hazards model. All statistical analyses were conducted using SPSS Statistics version 22.0 (IBM Corp., Armonk, New York) and GraphPad Prism software version 6.0 (GraphPad Sofetware, La Jolla, California). All reported *P*-values are 2-tailed, and *P*-value < 0.05 were considered to indicate statistical significance.

## Results

### Baseline characteristics

A total of 554 patients (72.2% male, mean age 59.8 ± 14.5 years) were included in this study. Baseline characteristics of patients divided by the presence of ES are shown in Table 1. For the entire study population, the proportions of dilated cardiomyopathy (DCM) and the hypertrophic cardiomyopathy (HCM) were 26.0% (144/554) and 5.6% (31/554), respectively. Besides, there were 8 cases with long QT syndrome, 6 patients with arrhythmogenic right ventricular cardiomyopathy (ARVC) and 2 with Brugada syndrome. Patients with prior MI accounted for 15.5% (86/554) and pre-implantation syncope accounted for 23.3% (129/554). A total of 199 (35.9%) patients had primary prevention indications for ICD therapy.

**Table 1:** Baseline characteristics of patients stratified by electrical storm (ES).

**Parameters**	**Whole population (*****n*** = **554)**	**ES (*****n*** = **76)**	**No-ES (*****n*** = **478)**	***P-*** **value**
Demographics
Age at implantation, years old	59.80 ± 14.48	62.08 ± 13.66	59.44 ± 14.59	0.141
Male sex, %	400 (72.2%)	54 (71.1%)	346 (72.4%)	0.810
NYHA class III-IV, %	209 (37.7%)	26 (34.2%)	183 (38.3%)	0.527
Primary prevention, %	199 (35.9%)	24 (31.6%)	175 (36.6%)	0.396
BMI	23.55 ± 3.31	24.00 ± 2.49	23.48 ± 3.42	0.212
Comorbidities, %
ICM	187 (33.8%)	28 (36.8%)	159 (33.3%)	0.602
DCM	144 (26.0%)	28 (36.8%)	159 (33.3%)	0.726
HCM	31 (5.6%)	1 (1.3%)	30 (6.3%)	0.104
Long QT syndrome	8 (1.4%)	0	8 (1.7%)	0.607
ARVC	6 (1.1%)	0	6 (1.3%)	1.000
Brugada syndrome	2 (0.36%)	0	2 (0.42%)	1.000
Prior MI	86 (15.5%)	12 (15.8%)	74 (15.5%)	0.945
Hypertension	129 (23.3%)	19 (25.0%)	110 (23.0%)	0.770
DM	45 (8.1%)	5 (6.6%)	40 (8.4%)	0.662
Pre-implantation syncope	129 (23.3%)	19 (25%)	110 (23%)	0.770
Valvular heart disease	13 (2.3%)	3 (3.9%)	10 (2.1%)	0.402
Echocardiography
LVEF, %	46.48 ± 14.80	46.66 ± 14.64	46.45 ± 14.83	0.860
LVEF group				0.757
≥40%	341 (61.6%)	48 (63.2%)	293 (61.3%)	
<40%	213 (38.4%)	28 (36.8%)	185 (38.7%)	
Medications, %
Beta-blockers	357 (64.4%)	47 (61.8%)	287 (64.8%)	0.795
ACEI or ARB	149 (26.9%)	23 (30.3%)	126 (26.4%)	0.488
Digoxin	128 (13.7%)	14 (18.4%)	62 (13.0%)	0.210
Amiodarone	165 (29.8%)	24 (31.6%)	141 (29.5%)	0.712

*ACEI, angiotensin-converting enzyme inhibitor; ARB, angiotensin receptor blocker; ARVC, arrhythmogenic right ventricular cardiomyopathy; BMI, body mass index; DCM, dilated cardiomyopathy; DM, diabetes mellitus; ES, electrical storm; HCM, hypertrophic cardiomyopathy; ICD, implantable cardioverter defibrillator; ICM, ischemic cardiomyopathy; LVEF, left ventricular ejection fraction; MI, myocardial infarction; NYHA, New York Heart Association*.

### ES episodes and treatment

ES was documented in a total of 76 patients, with an incidence of 13.7%, and 252 (45.5%) patients had impaired LVEF. The incidence of ES in preserved LVEF group and in impaired LVEF group were 13.1 and 14.1%, respectively, and the difference between two groups was not statistically significant. As shown in Table 2, a total of 242 episodes of ES were observed during follow up with a median episode of ES was 2 (range, 1–15) and about 75% (54/76) of ES patients experienced more than 3 episodes. The majority of ES episodes were attributed to VT events only (170/242, 70.2%) and a total of 2,095 VT events were observed including 1,422 slow VTs (140–170 bpm) and 673 fast VTs (171–210 bpm). The remaining episodes were due to VF (35/242, 14.5%) or a combination of VT and VF (37/242, 15.3%), with the VF episodes of 699. No ES episode was associated with syncope or required external resuscitation. ATP alone were used to treat 131 of 242 ES episodes (54.1%), and the remaining episodes were treated with ATP combined with shocks (81/242, 33.47%), shocks alone (22/242, 9.1%) or without therapy (8/242, 3.3%).

**Table 2:** Ventricular arrhythmias and device therapies of ES episodes during follow up.

**Characteristics**	**Overall ES episodes (*****n*** = **242)**
Ventricular arrhythmia types
VT only	170 (70.24%)
VT combined with VF	37 (15.28%)
VF only	35 (14.47%)
Device therapies of ES
With no device therapy	8 (3.30%)
ATP only	131 (54.13%)
ATP combined with shock	81 (33.47%)
Shock only	22 (9.09%)

*ATP, anti-tachycardia therapy; ES, electrical storm; VT, ventricular tachycardia; VF, ventricular fibrillation*.

### Risk of mortality analysis

**Figure 1:**
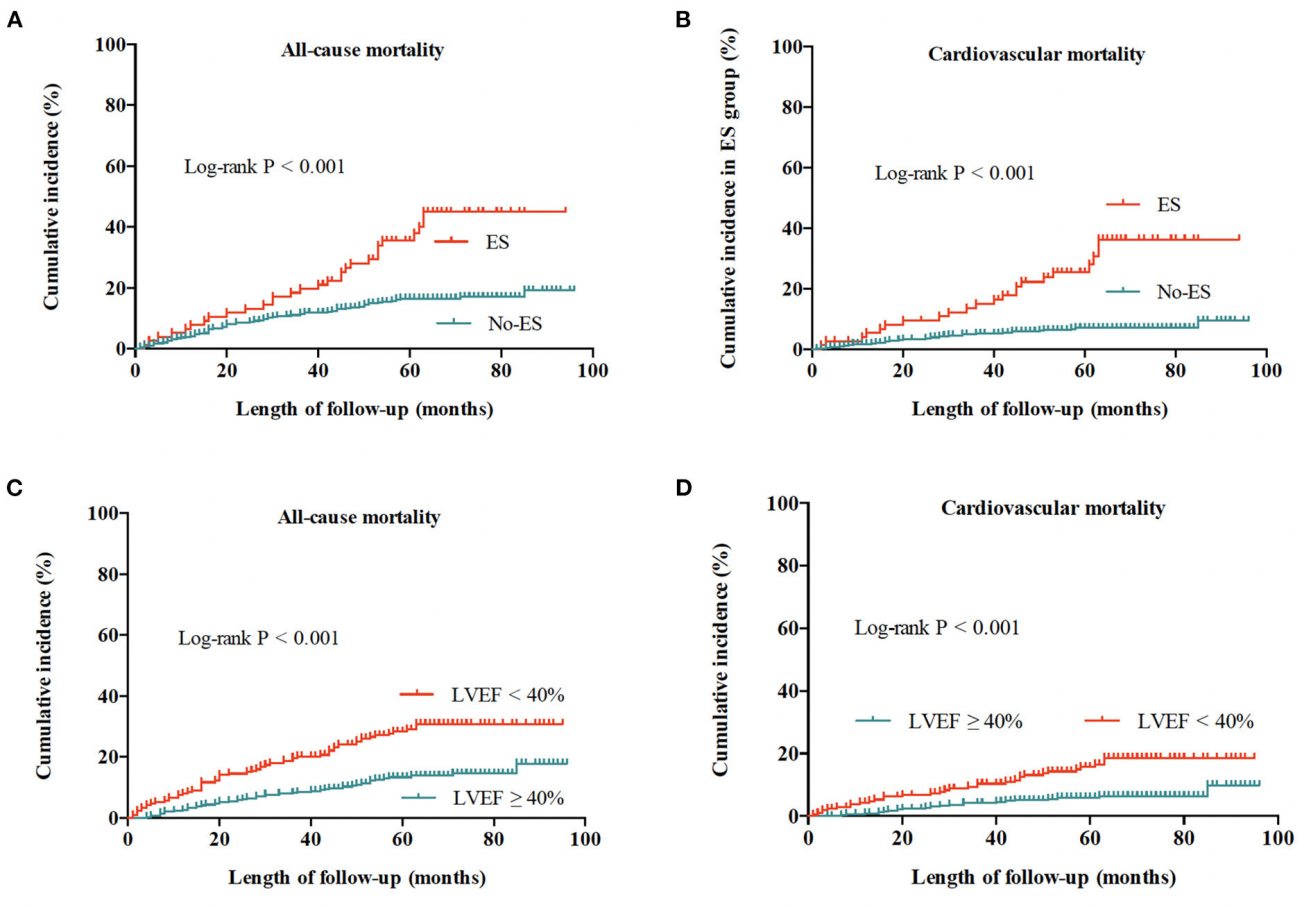
Cumulative incidences of all-cause and cardiovascular mortality for the whole population. **(A)** All-cause mortality for patients with ES and without ES. **(B)** Cardiovascular mortality for patients with ES and without ES. **(C)** All-cause mortality for patients with preserved LVEF (≥40%) and impaired LVEF (<40%). **(D)** Cardiovascular mortality for patients with preserved LVEF (≥40%) and impaired LVEF (<40%).

**Figure 2:**
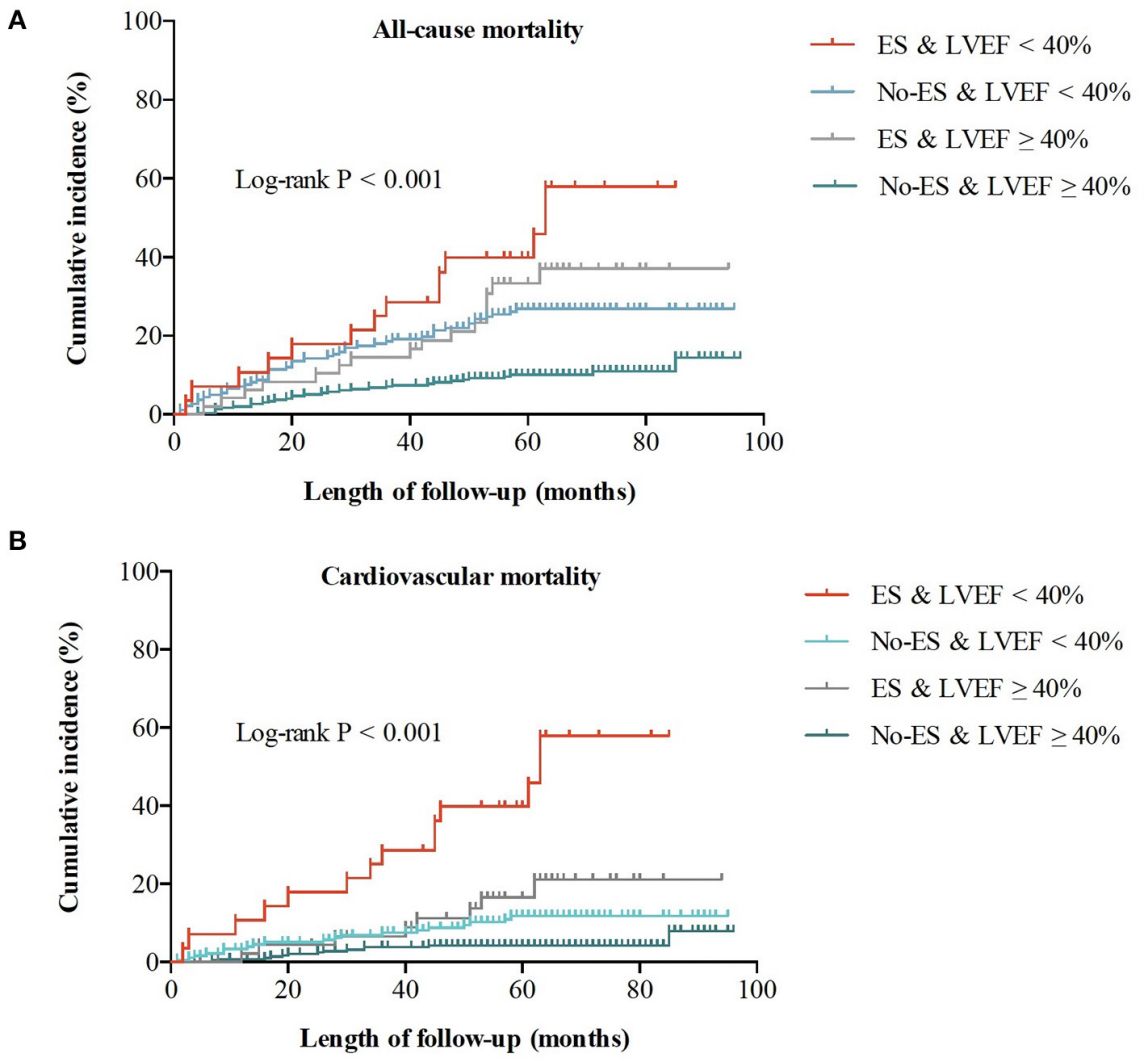
Cumulative incidences of all-cause and cardiovascular mortality for the whole population. **(A)** All-cause mortality for 4 groups. **(B)** Cardiovascular mortality for 4 groups.

During a median follow-up time (interquartile range) of 58 (52–71) months, a total of 109 patients died of all causes, with an all-cause mortality of 19.7%, and cardiovascular mortality of 9.7%. As shown in Figure 1, the risks of all-cause and cardiovascular death in patients without ES were both lower than risks in patients with ES (both log-rank *P* < 0.001), and patients with preserved LVEF had a better outcome when compared to patients with impaired LVEF (both log-rank *P* < 0.001). The Kaplan-Meier survival curves of the four groups in Figure 2 show that all-cause death risk and cardiovascular death risk were both highest in Group I (ES +LVEF < 40%), followed sequentially by Group III (ES + LVEF ≥ 40%), Group II (No-ES + LVEF < 40%) and Group IV (No-ES + LVEF ≥ 40%), and the differences were significant statistically (both log-rank *P*-values < 0.001).

### Effect of ES status and impaired LVEF on mortality

In the univariable Cox analysis shown in Table 3, the presence of ES was a predictor of all-cause and cardiovascular mortality (HR 2.65, 95%CI 1.74–4.05, *P* < 0.001; HR 4.85, 95% CI 2.81–8.34, *P* < 0.001, respectively). Similarly, compared to patients with preserved LVEF, impaired LVEF was also a risk factor of all-cause mortality and cardiovascular mortality (HR 2.33, 95% CI 1.64–3.41, *P* < 0.001; HR 2.79, 95% CI 1.61–4.82, *P* < 0.001, respectively). According to the results of univariable Cox regression, the parameters including ES, LVEF groups, age, ischemic cardiomyopathy, beta-blocker and digoxin were enrolled for multivariable Cox analysis for all-cause mortality and ES, LVEF groups and aging were included for cardiovascular mortality analysis. As shown in Table 4, the effects of ES and impaired LVEF were independently significant. The HR for ES was 2.40 (95% CI 1.57–3.68, *P* < 0.001) for all-cause death, and was 4.63 (95% CI 2.68–7.98, *P* < 0.001) for cardiovascular death. The HRs for impaired LVEF for all-cause death and cardiovascular death were 1.94 (95% CI 1.30–2.90, *P* = 0.001) and 2.56 (95% CI 1.47–4.44, *P* = 0.001), respectively.

**Table 3:** Univariable cox regression hazard ratios for all-cause and cardiovascular mortality.

**Variable**	**All-cause mortality**						**Cardiovascular mortality**					
	**ES (*****n*** = **76)**		**No-ES (*****n*** = **478)**		**All patients (*****n*** = **554)**		**ES (*****n*** = **76)**		**No-ES (*****n*** = **478)**		**All patients (*****n*** = **554)**	
	**HR (95% CI)**	***P-*** **value**	**HR (95% CI)**	***P-*** **value**	**HR (95% CI)**	***P-*** **value**	**HR (95% CI)**	***P-*** **value**	**HR (95% CI)**	***P-*** **value**	**HR (95% CI)**	***P-*** **value**
Age	1.05 (1.02–1.08)	0.003^*^	1.03 (1.02–1.05)	<0.001^*^	1.04 (1.02–1.05)	<0.001^*^	1.05 (1.01–1.09)	0.006^*^	1.02 (0.99–1.05)	0.137	1.03 (1.01–1.05)	0.004^*^
Gender	1.08 (0.48–2.42)	0.861	1.00 (0.61–1.63)	0.984	1.02 (0.67–1.55)	0.942	1.71 (0.58–5.07)	0.330	1.15 (0.52–2.56)	0.735	1.35 (0.71–2.57)	0.357
ES	NA		NA		2.65 (1.74–4.05)	<0.001^*^	NA		NA		4.85 (2.81–8.34)	<0.001^*^
LVEF <40%	1.64 (0.80–3.38)	0.179	2.69 (1.71–4.23)	<0.001^*^	2.33 (1.60–3.41)	<0.001^*^	3.29 (1.38–7.84)	0.007^*^	2.54 (1.25–5.14)	0.010^*^	2.79 (1.61–4.82)	<0.001^*^
ICM	1.27 (0.61–2.64)	0.523	1.81 (1.17–2.82)	0.008	1.67 (1.15–2.44)	0.008^*^	1.10 (0.46–2.61)	0.838	1.80 (0.89–3.60)	0.100	1.50 (0.88–2.58)	0.139
Beta-blocker	0.73 (0.34–1.55)	0.407	0.63 (0.40–1.00)	0.048^*^	0.64 (0.43–0.94)	0.024^*^	1.48 (0.60–3.64)	0.393	0.99 (0.49–1.98)	0.972	0.82 (0.48–1.42)	0.479
BMI	0.83 (0.71–0.97)	0.021^*^	0.97 (0.90–1.04)	0.359	0.96 (0.90–1.02)	0.169	0.87 (0.72–1.05)	0.134	1.00 (0.90–1.12)	0.995	0.99 (0.91–1.08)	0.792
Hypertension	1.06 (0.47–2.38)	0.888	1.12 (0.65–1.91)	0.687	1.06 (0.68–1.65)	0.810	1.11 (0.43–2.84)	0.825	1.49 (0.70–3.14)	0.298	1.33 (0.74–2.39)	0.335
DM	0.44 (0.05–2.88)	0.357	0.82 (0.40–1.71)	0.598	1.00 (0.51–1.98)	1.000	0.53 (0.07–3.97)	0.537	0.71 (0.17–2.98)	0.640	1.53 (0.48–4.90)	0.476
Pre-implantation syncope	1.06 (0.94–2.38)	0.888	1.46 (0.52–1.54)	0.687	1.06 (0.68–1.65)	0.810	1.11 (0.43–2.84)	0.825	0.67 (0.32–1.42)	0.298	1.33 (0.74–2.39)	0.335
ACEI/ARB	0.70 (0.34–1.48)	0.354	0.77 (0.48–1.24)	0.280	0.74 (0.49–1.1)	0.737	0.72 (0.30–1.71)	0.454	1.26 (0.59–2.67)	0.548	1.36 (0.77–2.40)	0.285
Digoxin	1.42 (0.68–3.13)	0.659	2.16 (1.28–3.66)	0.004^*^	2.04 (1.31–3.20)	0.002^*^	1.06 (0.36–3.13)	0.918	2.04 (0.88–4.76)	0.095	1.75 (0.90–3.45)	0.092
Amiodarone	0.83 (0.40–1.74)	0.615	0.59 (0.34–1.01)	0.058	0.75 (0.05–1.16)	0.194	0.98 (0.40–2.40)	0.959	0.53 (0.22–1.30)	0.165	1.40 (0.75–2.61)	0.295

*ACEI, angiotensin-converting enzyme inhibitor; ARB, angiotensin receptor blocker; BMI, body mass index; DM, diabetes mellitus; ES, electrical storm; ICM, ischemic cardiomyopathy; LVEF, left ventricular ejection fraction; NA, not available*.

* *Statistical significance, P < 0.05*.

**Table 4:** Multivariable cox regression analysis hazard ratios of all-cause and cardiovascular mortality for the whole population.

**Variable**	**All-cause mortality**		**Cardiovascular mortality**	
	**HR (95% CI)**	***P-*** **value**	**HR (95% CI)**	***P-*** **value**
ES vs. No ES	2.40 (1.57–3.68)	<0.001^*^	4.63 (2.68–7.98)	<0.001^*^
LVEF <40% vs. LVEF ≥ 40%	1.94 (1.30–2.90)	0.001^*^	2.56 (1.47–4.44)	0.001^*^
ES and LVEF
No ES, LVEF ≥40% (ref)
No ES, LVEF <40%	2.37 (1.48–3.78)	<0.001^*^	2.48 (1.22–5.04)	0.012^*^
ES, LVEF ≥ 40%	3.42 (1.87–6.25)	<0.001^*^	4.31 (1.78–10.41)	0.001^*^
ES, LVEF <40%	4.17 (2.16–8.06)	<0.001^*^	11.91 (5.55–25.56)	<0.001^*^
Age	1.03 (1.02–1.05)	<0.001^*^	1.02 (1.00–1.05)	0.026^*^
Ischemic cardiomyopathy	1.22 (0.82–1.80)	0.326		
Beta-blocker	0.65 (0.43–0.97)	0.036^*^		
Digoxin	1.21 (0.75–1.95)	0.432		

*ACEI, angiotensin-converting enzyme inhibitor; ARB, angiotensin receptor blocker; ES, electrical storm; HR, hazard ratio; LVEF, left ventricular ejection fraction*.

* *Statistical significance, P < 0.05*.

Additionally, as shown in Table 4, compared to patients with preserved LVEF in the absence of ES, the risks of all-cause mortality and cardiovascular mortality in ES patients with impaired LVEF were both increased significantly (HR 4.17, 95% CI 2.16–8.06, *P* < 0.001; HR 11.91, 95% CI 5.55–25.56, *P* < 0.001, respectively). The mortality risks in patients with ES and preserved LVEF, and in patients with preserved LVEF and without ES were both significantly higher. For patients with ES and preserved LVEF, the HR for all-cause death was 3.42 (95% CI 1.87–6.25, *P* < 0.001) and the HR for cardiovascular death was 4.31 (95% CI 1.78–10.41, *P* = 0.001). The HRs for patients with preserved LVEF and without ES were 2.37 (95% CI 1.48–3.78, *P* < 0.001) and 2.48 (95% CI 1.22–5.04, *P* = 0.012), respectively. Of note, the HRs for ES combined with impaired LVEF were both highest among the four groups.

### Effect of ES status on mortality relative to LVEF impairment

**Figure 3:**
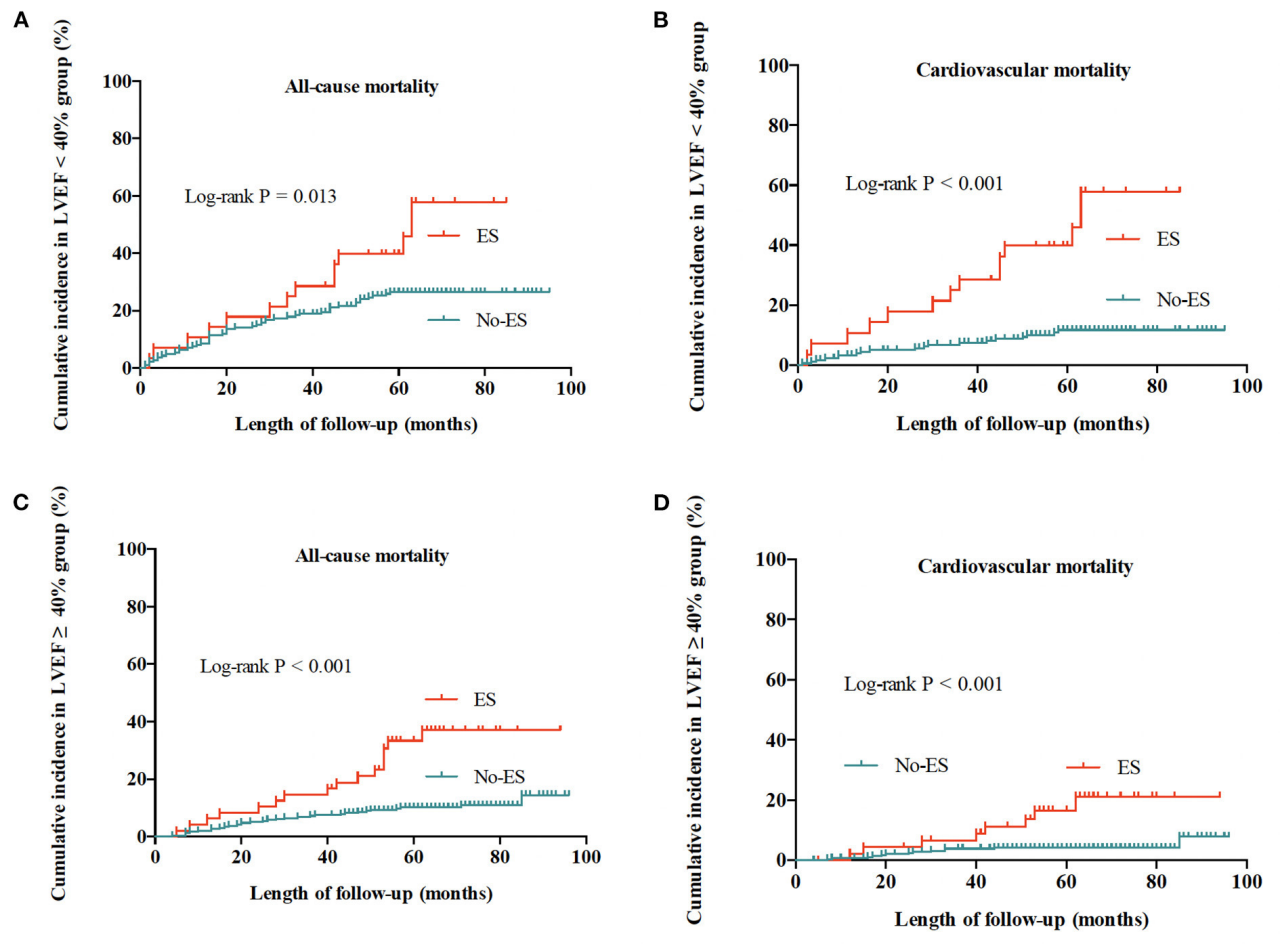
Cumulative incidences of all-cause and cardiovascular mortality for patients with preserved LVEF (≥40%) and impaired LVEF (<40%). **(A)** All-cause mortality for patients with impaired LVEF. **(B)** Cardiovascular mortality for patients with impaired LVEF. **(C)** All-cause mortality for patients with preserved LVEF. **(D)** Cardiovascular mortality for patients with preserved LVEF.

In patients with preserved LVEF and in patients with impaired LVEF, ES both significantly increased the mortality risks of patients when compared to No-ES patients (shown in Figure 3). As shown in Table 5, for patients with preserved LVEF, the risks of all-cause mortality and cardiovascular mortality in ES patients were both significantly higher than risks in No-ES patients (HR 3.33, 95% CI 1.81–6.13, *P* < 0.001; HR 4.16, 95% CI 1.70–10.13, *P* = 0.002, respectively); for patients with impaired LVEF, the HR for all-cause mortality was 1.84 (95% CI 1.01–3.37, *p* = 0.034) and the HR for cardiovascular mortality was 4.86 (95% CI 2.39–9.86, *P* < 0.001).

**Table 5:** Selected comparisons among subgroups.

**Comparison**	**Adjusted HR (95% CI)** **for all-cause mortality**	***P-*** **value**	**Adjusted HR (95% CI)** **for cardiovascular mortality**	***P-*** **value**
LVEF ≥ 40%: ES vs. No ES	3.33 (1.81–6.13)	<0.001^*^	4.16 (1.70–10.13)	0.002^*^
LVEF <40%: ES vs. No ES	1.84 (1.00–3.37)	0.034^*^	4.86 (2.39–9.86)	<0.001^*^
ES: LVEF <40% vs. ≥ 40%	1.14 (0.54–2.38)	0.735	2.54 (1.05–6.12)	0.038^*^
No-ES: LVEF <40% vs. ≥ 40	2.26 (1.40–3.63)	0.001^*^	2.54 (1.25–5.14)	0.010^*^

*ES, electrical storm; HR, hazard ratio; LVEF, left ventricular ejection fraction*.

* *Statistical significance, P < 0.05*.

### Effect of LVEF impairment on mortality relative to ES status

**Figure 4:**
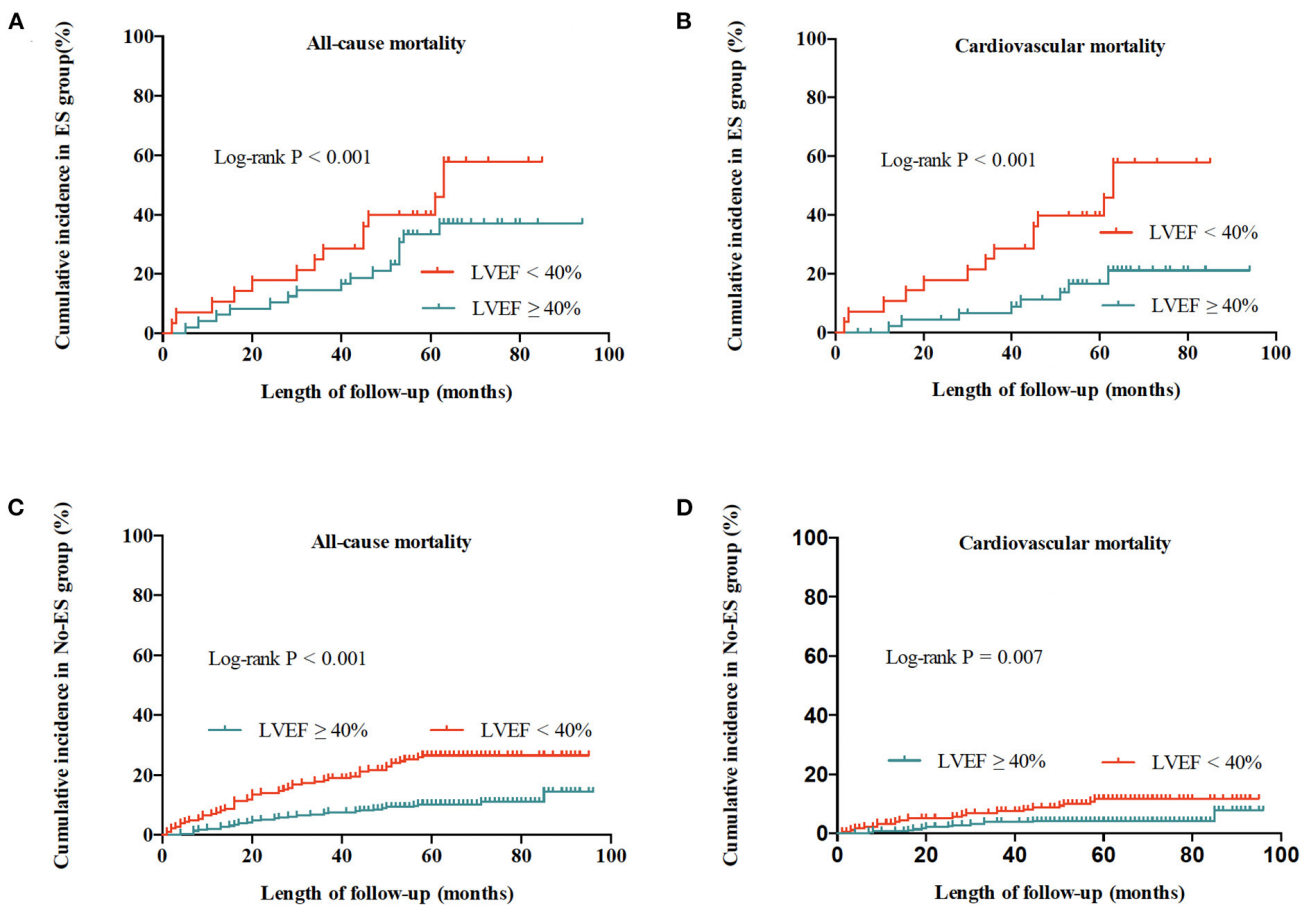
Cumulative incidences of all-cause and cardiovascular mortality for patients with ES and without ES. **(A)** All-cause mortality for patients with ES. **(B)** Cardiovascular mortality for patients with ES. **(C)** All-cause mortality for patients without ES. **(D)** Cardiovascular mortality for patients without ES.

As shown in Table 5 and Figure 4, in the No-ES group, as compared to patients to the preserved LVEF group, impaired LVEF was associated with increased risks of all-cause mortality and cardiovascular mortality (HR 2.26, 95% CI 1.40–3.63, *p* = 0.001; HR 2.54, 95% CI 1.25–5.14, *P* = 0.010, respectively). In ES group, as compared to the preserved LVEF group, impairment of LVEF increased the risk of cardiovascular death significantly (HR 2.54, 95% CI 1.05–6.12, *P* = 0.038). However, patients with impaired LVEF showed no deleterious effect on all-cause mortality risk when compared to those with preserved LVEF (HR 1.14, 95% CI 0.54–2.38, *P* = 0.735).

Additionally, multivariable Cox analysis demonstrated that aging was the only other statistically significant predictor of all-cause and cardiovascular mortality in patients with ICD (HR 1.03, 95% CI 1.02–1.05, *P* < 0.001; HR 1.02, 95% CI 1.00–1.05, *P* = 0.026, respectively), and beta-blocker use was an independent protective factor for all-cause mortality (HR 0.65, 95% CI 0.43–0.97, *P* = 0.036).

## Discussion

The main findings of the present study are that both ES and impaired LVEF act as independent predictors for mortality in patients with ICD, and the interaction of ES and impaired LVEF significantly amplified the deleterious effects of each of these distinct entities. In patients with preserved LVEF, the risk of all-cause mortality and cardiovascular mortality for ES vs. No-ES were increased by more than 3-folds and more than 4-folds, respectively. In in patients with impaired LVEF, ES was associated with 84% increased all-cause mortality and more than 4-fold increase of cardiovascular death. When compared to patients with a preserved LVEF, impaired LVEF acted as an effect modifier that significantly increased the all-cause death risk and cardiovascular mortality risk both by more than 2-folds in patients without ES. In the presence of ES, the deleterious effect of impaired LVEF seemed to be confined to the cardiovascular death risk, which was substantially increased by more than 2-folds, but it did not show a consistent effect on the all-cause mortality risk.

### ES prevalence and effect on risk of mortality

ES is not rare in clinical practice, but its real prevalence in ICD recipients varies widely in current literature, ranging from 2.9 to 40% (3, 5, 6, 8). The incidence of ES in our population was 13.7%, and this finding is in fact close to the reported 10% in the study of Credner et al. (9), 14% reported by Arya et al. (6) and the incidence of 18.9% reported by Gztzoulis et al. (10). The difference in ES prevalence may be due to the differences in clinical characteristics among the different populations, the different definitions of ES, the underlying heart disease, and the length of follow-up. Interestingly, in our study there was no difference in prevalence of ES between impaired LVEF group and preserved LVEF group.

The survival curves in our study show a significant difference in mortality between the patients with and without ES, and multivariable Cox analysis revealed that ES was an independent predictor for increased risk of all-cause mortality (HR 2.40, 95% CI 1.57–3.68, *p* < 0.00), which is consistent with the findings of previous studies (3, 7, 10, 11). In 2001, Exner et al. (7) reported that in the AVID study, ES was associated independently with all-cause mortality and displayed a more than 2-fold higher risk. Similarly, Sesselberg et al. (11) reported that in the MADIT-II study, patients who experienced ES had a significantly higher risk of death, with a higher HR for ES of 17.8 during the follow-up. More importantly, a meta-analysis of a large population including 13 previous studies has confirmed the effect of ES as an independent strong predictor for mortality in ICD patients (risk ratio 3.15) (1). Consistently, through a long-term follow-up, our findings provide additional support to the knowledge that ES is associated with a significant increase in all-cause mortality risk of ICD recipients, regardless of the other variables (HR 2.26, 95% CI 1.40–363, *P* = 0.001). Additionally, we found that the effect of ES on the risk of cardiovascular death was more substantial, with HR of 4.63 (95% CI 2.68–7.98, *P* < 0.001), which was a new finding regarding to the deleterious effects of ES on patients.

### Effect of LVEF impairment on risk of mortality

It has been well known that reduced LVEF is associated with poor outcome in patients with ICD. In 2001, the results of AVID trial showed that LVEF was a significant risk factor for mortality, independent of ES and other prognostic variables (7). In 2018, Extramiana et al. (12) demonstrated that reduced LVEF remains the major influencing factor in patients with ICD for secondary prevention. Moreover, a meta-analysis of 72 studies confirmed the effects of reduced LVEF as an independent predictor for poor outcome in ICD patients (2). Consistent with previous reports, our findings show that impaired LVEF acts as an independent indicator of all-cause death in patients (HR 1.94, 95% CI 1.30–2.90, *P* = 0.001). Additionally, similar to ES, we also found that impaired LVEF was a significant risk predictor for cardiovascular death (HR 2.56, 95% CI 1.47–4.44, *P* = 0.001).

### Interaction between ES and LVEF impairment on mortality

As discussed above, initial studies have shown that ES and LVEF are the two major risk factors of mortality in ICD recipients (3, 7, 10, 11), but whether ES is an independent causal factor or just an epiphenomenon of severely impaired LVEF is unclear. It should be pointed out that the systolic function of the whole populations in the current literature was already severely hampered, with LVEF ranging from 22 to 41% (1). According to this background, none of the previous studies has focused on the interaction between ES and LVEF impairment on mortality in patients with ICD. Attributed to the high proportion of ICD recipients for secondary prevention in our study (64%), different from the other the other studies (3, 7, 10, 11), 61% patients in our study had preserved LVEF (≥40%), and therefore, we were able to investigate whether the deleterious effect of ES on mortality is an epiphenomenon of LVEF impairment. Our results showed that not only both ES and impaired LVEF acted as strong risk factors of mortality for ICD recipients, the coexistence of ES and impaired LVEF further increases the mortality risk synergistically. Compared with the group IV (No-ES and preserved LVEF), which had the most favorable outcome, both the all-cause and cardiovascular mortality of group I (ES and impaired LVEF) were much higher (HR 4.17, 95% CI 2.16–8.06, *P* < 0.001; HR 11.91, 95% CI 5.55–25.56, *P* < 0.001, respectively), and were highest among the four groups. For patients with impaired LVEF, the presence of ES significantly increased the risk of mortality, irrespective of all-cause and cardiovascular death, which is consistent with the effect of ES on the whole population and on patients with preserved LVEF. Similarly, we found that when compared with preserved LVEF, impaired LVEF increased the risk of cardiovascular death in patients with ES, although it had no deleterious effect on the all-cause mortality.

Although we found that the effect of ES on mortality is independent of impaired LVEF, little is known regarding to the potential mechanisms. A series of previous studies indicated that progressive deterioration of cardiac function resulting from frequent shocks through myocardial damage, inflammation and electrical remodeling (13), hampered cardiac contractility due to long-term high VT/VF burden and systemic toxicity from high dose antiarrhythmic drug therapy could all be associated with the adverse outcome of ES patients (14, 15). Our results suggest that prevention of LVEF impairment after an initial ES event and modification of ES substrate are both essential in order to improve the prognosis of patients. To prevent the further deterioration of LVEF, treatment focused on the underlying heart disease and optimal pharmacological regimen, or cardiac resynchronization therapy could be considerd (16). For the optimal management of ES, consensus has not been achieved yet. Recent studies have demonstrated that patients with ES may have significant benefit from more effective pharmacological therapy, such as the combination of amiodaraone and propranolol (17) and more aggressive treatment, such as prophylactic catheter ablation for ventricular tachyarrhythmias (18) and sympathetic blockade (19).

### Limitations

Our study has several limitations. First, as a retrospective analysis, our study suffers from limitations typically associated with this type of design and there may be a potential selection bias since all of our patients received Biotronic ICDs with home monitoring system. Second, data analyzed were collected before ICD implantation and serial reassessment during follow-up was not available, especially the changes of LVEF. The goal of this study was to investigate the interaction between ES and LVEF impairment on the mortality of ICD recipients and there may be a relationship between the trend of LVEF during long-term follow up whether improved, deteriorate for just keeping stable. We believe that it would be better to get the latest value of LVEF. However, our study was retrospective without requiring patient to undergo echocardiography at specific time, so it was unavailable to collect the data. In the future studies, it should be further investigated whether improvement of LVEF results in a better outcome for patients in the presence of ES. Third, the rates of beta-blocker and ACEI/ARB at baseline in our study were low and there was a large gap between our results and the recommendations in guidelines. The reasons may be various. Our patients were enrolled between 2010 and 2014, and there was a lack of awareness of integrated management among Chinese physicians at that time. Besides, physicians would like to make an adjustment of medical therapy at 1-month follow up after ICD implantation when the patients' conditions were stable. The rates of beta-blocker and ACEI/ARB at that time were 78.7 and 46.0%, respectively, showing a significant increase that at the baseline. In addition, restricted by the retrospective design, we have no access to the parameters such as the blood pressures, heart rates, level of blood potassium and the renal function, which also could affect the medical therapy. Forth, the underlying heart diseases in study included ICM, DCM and as well as some cases of HCM, ARVC, Brugada and long QT syndrome, which increased the sample heterogeneity of our population. Fifth, by virtue of the smaller group of ES patients and the Chinese characteristics in population and management, even if statistical significance was reached, our findings should be substantiated by clinical studies with a larger sample size of ES patients and better representativeness in the future.

## Conclusion

Both ES and impaired LVEF are independent predictors of mortality risk in ICD recipients. The presence of both ES and impaired LVEF in patients significantly amplifies the deleterious effects of each distinct condition.

## Data availability statement

The original contributions presented in the study are included in the article/supplementary material, further inquiries can be directed to the corresponding authors.

## Ethics statement

The studies involving human participants were reviewed and approved by Ethics Committee of Fuwai Hospital. The patients/participants provided their written informed consent to participate in this study.

## Author contributions

The study was designed by SZ and ZZ. Material preparation, data collection, and analysis were performed by ZZ, SZ, and XL. The first draft of the manuscript was written by ZZ. All authors reviewed, edited, approved the final version of the manuscript, and contributed to design this study.

## Funding

This research was supported by National Natural Science Foundation of China (81470466) and Natural Key Clinical Specialty Construction Project (No. 2020-QTL-009), which only provided financial support but did not intervene in the designing, performing, data interpretation, and publication of the study.

## Conflict of interest

The authors declare that the research was conducted in the absence of any commercial or financial relationships that could be construed as a potential conflict of interest.

## Publisher's note

All claims expressed in this article are solely those of the authors and do not necessarily represent those of their affiliated organizations, or those of the publisher, the editors and the reviewers. Any product that may be evaluated in this article, or claim that may be made by its manufacturer, is not guaranteed or endorsed by the publisher.

## Abbreviations

ACEI: angiotensin-converting enzyme inhibitor
ARB: angiotensin receptor blocker
ATP: anti-tachycardia pacing
ARVC: arrhythmogenic right ventricular cardiomyopathy
BMI: body mass index
DCM: dilated cardiomyopathy
DM: diabetes mellitus
ES: electrical storm
HCM: hypertrophic cardiomyopathy
ICD: implantable cardioverter defibrillator
ICM: ischemic cardiomyopathy
LVEF: left ventricular ejection fraction
MI: myocardial infarction
NYHA: New York Heart Association
VT: ventricular tachycardia
VF: ventricular fibrillation.
